# Olfactory and Gustatory Disturbances as Early Indicators of Lung Cancer in Patients with Sleep Disorders: A Retrospective Cohort Study from the TriNetX US Collaborative Networks

**DOI:** 10.7150/ijms.106014

**Published:** 2025-01-13

**Authors:** Ru-Yin Tsai, Chi-Chung Ho, Jhen-You Hu, Yao Hsiao, Hung-En Huang, James Cheng-Chung Wei

**Affiliations:** 1 Department of Anatomy, Faculty of Medicine, Chung Shan Medical University, Taichung, Taiwan.; 2 Department of Medical Education, Chung Shan Medical University Hospital, Taichung, Taiwan.; 3 Department of Physical Medicine and Rehabilitation, Chung Shan Medical University Hospital, Taichung, Taiwan.; 4 Department of Physical Medicine and Rehabilitation, Chung Shan Medical University, Taichung, Taiwan.; 5 Taichung Municipal Taichung Girls' Senior High School, Taichung, Taiwan.; 6 School of Medicine, Chung Shan Medical University, Taiwan.; 7 Center for Health Data Science, Department of Medical Research, Chung Shan Medical University Hospital, Taichung, Taiwan.; 8 Institute of Medicine, Chung Shan Medical University, Taichung, Taiwan.; 9 Department of Allergy, Immunology & Rheumatology, Chung Shan Medical University Hospital, Taichung, Taiwan.; 10 Graduate Institute of Integrated Medicine, China Medical University, Taichung, Taiwan.

**Keywords:** Melatonin, Anosmia, Dysgeusia, Hormonal Changes, Circadian Rhythm

## Abstract

**Background:** Olfactory and gustatory disturbances are commonly overlooked symptoms but may be linked to various health conditions, including cancer. Emerging evidence suggests that these sensory impairments could be early indicators of lung cancer, particularly in individuals with sleep disorders, a group already at elevated cancer risk due to factors like circadian disruption and hormonal changes.

**Objective:** To evaluate whether olfactory and gustatory disturbances can serve as early markers for lung cancer in patients with sleep disorders.

**Methods:** A retrospective cohort study was performed using data from the TriNetX database, spanning January 1, 2016, to June 30, 2024. Propensity score matching (1:1) was used to balance baseline characteristics between patients with olfactory and gustatory disturbances and a control group without these disturbances. Hazard ratios (HR) and 95% confidence intervals (CI) were calculated to assess lung cancer risk, with follow-up lasting up to 60 months.

**Results:** After matching, 13,294 patients with olfactory and gustatory disturbances and 13,294 control patients were included. The analysis revealed a significantly higher risk of lung cancer in the disturbance group (HR = 1.431, 95% CI: 1.014-2.021). Subgroup analysis indicated that the risk was particularly elevated in patients over 50, males, and those with dorsalgia. COVID-19 infection did not have a significant impact on lung cancer risk in this population.

**Conclusion:** Olfactory and gustatory disturbances may serve as early markers for lung cancer, particularly in older patients and males with sleep disorders. These findings suggest the potential for using sensory impairments in early cancer detection strategies.

## Introduction

Lung cancer remains one of the leading causes of cancer-related mortality worldwide, with early detection playing a crucial role in improving patient outcomes. Despite advancements in diagnostic technologies, identifying reliable early markers for lung cancer in high-risk populations remains a significant challenge. Recent research has suggested that sensory impairments, such as olfactory and gustatory disorders, may be linked to various types of cancers 1. These disorders, often overlooked in clinical assessments, could provide valuable insights into the pathophysiological changes occurring in individuals at an elevated risk of malignancies 2, particularly in the context of sleep disorders.

Sleep disorders, especially those prevalent among postmenopausal women 3, 4, have been associated with disruptions in circadian rhythms and reduced melatonin levels 4. These factors not only affect sleep quality but also have been implicated in the increased risk of various cancers, including lung cancer 5-7. Melatonin, a key hormone regulating sleep-wake cycles, also influences cellular processes such as DNA repair, oxidative stress, and immune response, which are critical in cancer development 8-10. Given that olfactory and gustatory functions are also modulated by circadian rhythms and melatonin levels 11, 12, these sensory impairments may serve as early indicators of cancer in individuals with sleep disorders.

Olfactory and gustatory disorders are frequently reported symptoms among patients with lung cancer^13^, ^14^, yet their potential role as early markers remains underexplored. Previous studies have shown that the presence of olfactory-related receptors on the membranes of lung cancer cells could indicate a biological link between these sensory disturbances and tumor development 15-17. Despite these findings, the clinical significance of olfactory and gustatory dysfunctions in identifying high-risk individuals for lung cancer remains unclear.

This study utilizes the TriNetX network, a global health research database, to conduct a retrospective cohort analysis exploring the link between olfactory and gustatory disorders and lung cancer risk in individuals with sleep disorders. By analyzing data from a large and diverse patient population, we aim to assess whether sensory impairments can serve as early indicators of lung cancer. This research seeks to enhance understanding of these dysfunctions as predictive markers and their potential use in clinical settings for early detection and intervention.

## Methods

### Study design and data source

The retrospective cohort study utilized aggregated data from TriNetX, a global health research network that provides access to electronic medical records (EMR) from a wide range of healthcare organizations (HCOs) 18. TriNetX employs a standardized framework to evaluate data quality, focusing on metrics such as conformance, completeness, and plausibility. Data extraction and analysis took place in September 2024, using the US collaborative network subset of TriNetX, which included 63 HCOs. The study period was defined from January 1, 2016, to June 30, 2024, to ensure a focused and relevant analysis.

### Ethics statement

TriNetX received a waiver from the Western Institutional Review Board (WIRB) as it only provides aggregated counts and statistical summaries of de-identified data. Chung Shan Medical University Hospital (CSMUH), as a member of TriNetX's Health Care Organization (HCO) network, has access to this de-identified data via the TriNetX platform. The Institutional Review Board of CSMUH also granted approval for the use of TriNetX data in this study (Approval No: CS2-21176). The study follows the guidelines of the Reporting of studies Conducted using Observational Routinely collected health Data (RECORD) Statement for cohort studies.

### Study subjects

**Figure 1:**
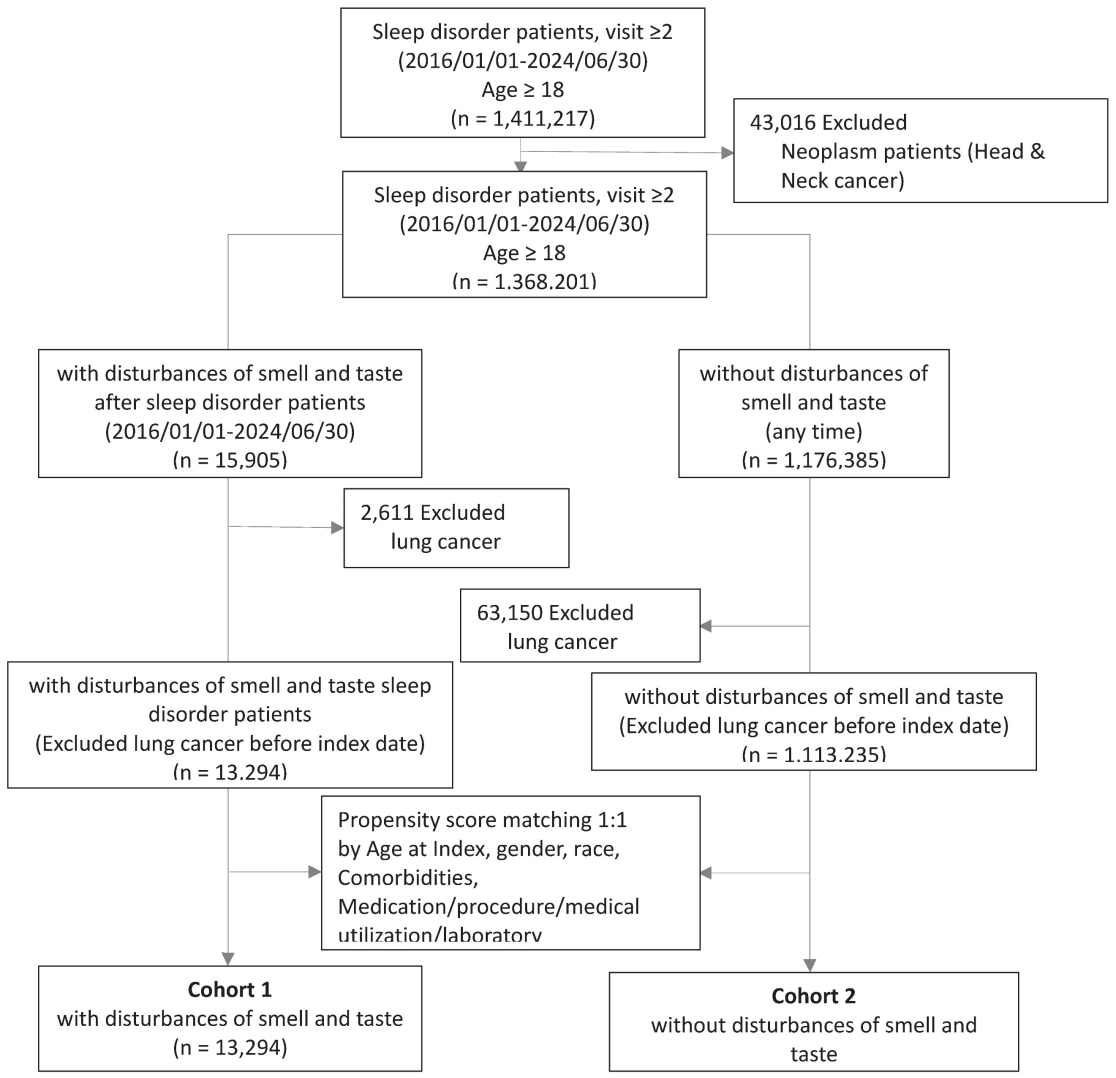
Flow chart of cohort construction.

The cohort construction flowchart (Figure 1) indicates that a total of 1,411,217 participants were registered from January 1, 2016, to June 30, 2024. The inclusion criteria required patients to have had more than two visits for sleep disorders (please refer to supplement material table S1 for detailed codes) during the specified period and to be at least 18 years of age. Patients with a history of cancers related to the nasal, pharyngeal, or laryngeal regions (ICD10: C10, C11, C13, C14.0, C14.8, C31.8, C32.8, C41.0, C49.0, C76.0, D02.0, D00.00, D00.08, D14.0, D14.1, D21.0, D49.1) and those with a history of tumors with uncertain behavior (ICD10: D36.7, D49.2, D49.89) were excluded (please refer to supplement material table S2 for detailed codes). Ultimately, 1,368,201 participants were included in the study cohort, with 15,905 individuals having developed olfactory and gustatory dysfunction (please refer to supplement material table S3 for detailed codes) after their sleep disorder diagnosis, and 1,113,235 individuals having no occurrence of such dysfunctions. Both groups excluded patients who had lung cancer before the index date.

In our cohort, we applied propensity score matching (1:1) based on factors such as age at index date, race, gender, comorbidities, medication use, and laboratory data. After matching, 13,294 participants with olfactory and gustatory dysfunction were selected, along with 13,294 controls without such dysfunctions, for further analysis. These subjects were followed for up to 5 years from the index date to estimate the risk of developing lung cancer.

### Covariates

To adjust for baseline differences between the two groups, we considered factors that could potentially influence lung cancer risk and included the following covariates: demographic characteristics (age at index, gender, and race). The comorbidities listed at baseline in this study included acute myocardial infarction (ICD10: I21), peripheral vascular disease (ICD10: I73), cerebrovascular disease (ICD10: I60-I69), chronic obstructive pulmonary disease (ICD10: J44), peptic ulcer (ICD10: K27), liver disease (ICD10: K76.9), diabetes mellitus (ICD10: E08-E13), chronic kidney disease (ICD10: N18.9), depression (ICD10: F32), anxiety (ICD10: F41), Alzheimer's disease (ICD10: G30), chronic pain syndrome (ICD10: G89.4), obesity (ICD10: E66), and COVID-19 (ICD10: U07.1).

We also utilized physical examination and laboratory test results to capture the differences in cancer markers between the two groups with abnormal values. The physical examination factor included BMI (overweight, ≥ 30 kg/m²). Laboratory tests analyzed in this study included carcinoembryonic antigen [mass/volume] in serum or plasma (≥ 5 ng/mL), alpha-fetoprotein [mass/volume] in serum, plasma, or blood (≥ 20 ng/mL), cancer antigen 125 [units/volume] in serum, plasma, or blood (≥ 35 U/mL), cancer antigen 19-9 [units/volume] in serum or plasma (≥ 37 U/mL), cancer antigen 15-3 [units/volume] in serum or plasma (≥ 30 U/mL), prostate-specific antigen [mass/volume] in serum or plasma (≥ 10 U/mL), and beta-subunit chorionic gonadotropin [units/volume] in serum, plasma, or blood (≥ 25 mIU/mL).

### Statistical analyses

To minimize the impact of confounding factors, we used propensity score matching to create study groups with similar baseline characteristics. Utilizing the built-in functionality of TriNetX, we matched the two groups at a 1:1 ratio, considering factors such as age, race, gender, comorbidities, medication use, and laboratory data. Standardized differences (Std diff) were used to assess the balance of baseline characteristics post-matching, with a Std diff < 0.1 generally indicating minimal differences.

We initiated follow-up from the first day after the assessment and continued for up to 60 months, calculating hazard ratios (HR) for lung cancer occurrence. The proportional hazards assumption was tested using the built-in generalized Schoenfeld method on the TriNetX platform. If the assumption was violated, hazard ratios for different time intervals were computed separately. In all analyses, a 95% confidence interval (95% CI) was used to determine statistical significance. The Kaplan-Meier method was employed to calculate survival probabilities, with statistical significance defined as a *P*-value < 0.05.

## Results

### Baseline characteristics of the study subjects

The baseline demographic information, comorbidities, healthcare utilization, medication use, and laboratory test results for both the influenza vaccine group and the control group are outlined before and after propensity score matching (Table 1). Following the matching process, the standardized differences across all characteristics between the two groups were less than 0.1, indicating minimal variation in baseline characteristics. Notably, COVID-19-related factors were also successfully matched, demonstrating that the matching procedure effectively minimized potential confounding variables, thereby enhancing the reliability of the subsequent analysis.

### Lung cancer incidence in patients with olfactory and gustatory disorders

**Figure 2:**
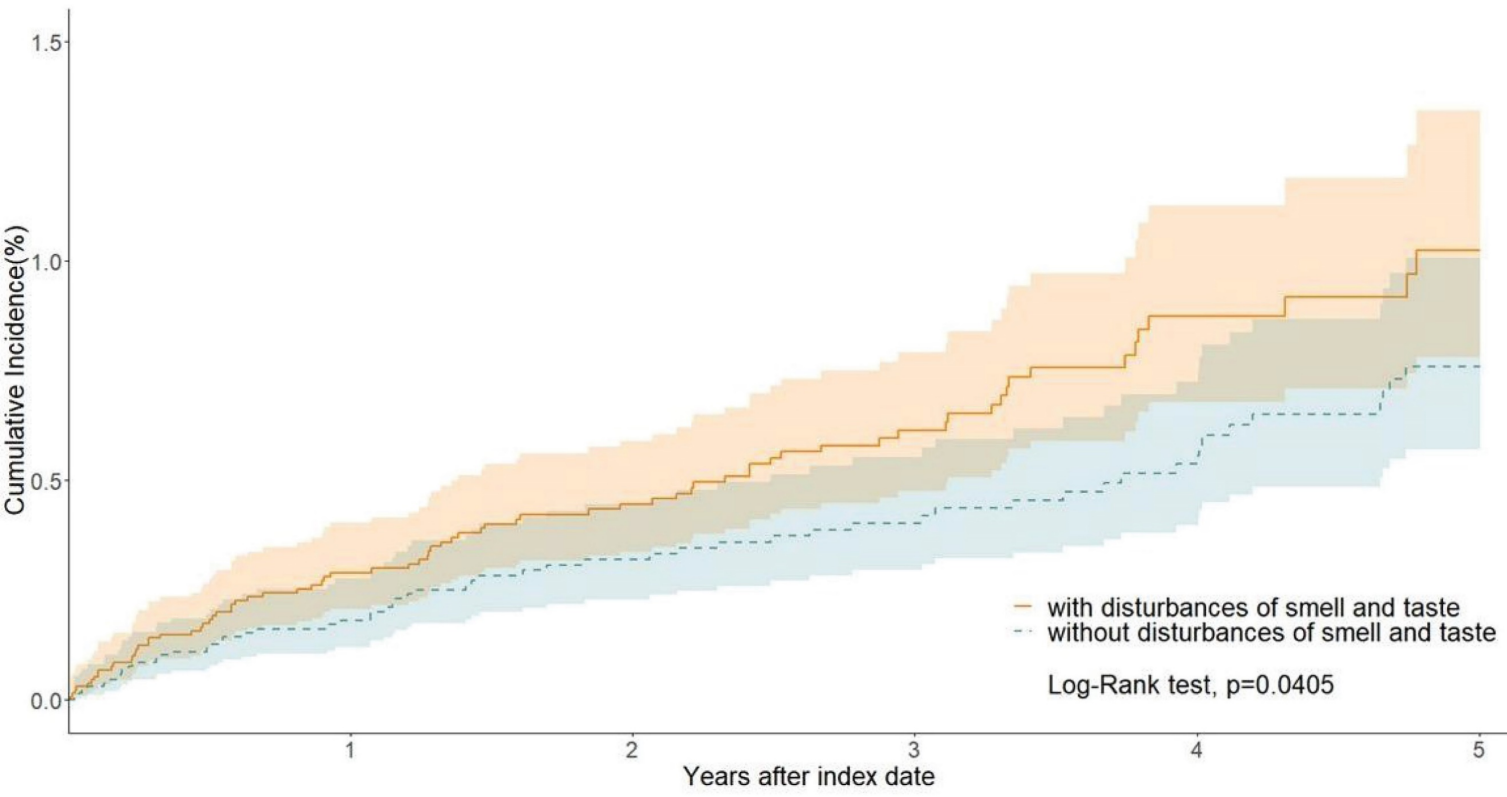
Kaplan-Meier curves of lung cancer.

We estimated the risk of lung cancer in patients with olfactory and gustatory disorders (Cohort 1) compared to the control group without these disorders (Cohort 2). Over the long-term follow-up period, patients with olfactory and gustatory disorders exhibited a significantly increased risk of developing lung cancer, with a hazard ratio (HR) of 1.431 (95% CI: 1.014-2.021). This indicates that olfactory and gustatory disorders have a significant impact on the increased risk of lung cancer (Table 2). Kaplan-Meier curves demonstrate a significant difference in the probability of smell and taste disturbance incidence between the two cohorts (p = 0.0405; Figure 2).

### Subgroup analyses

**Figure 3:**
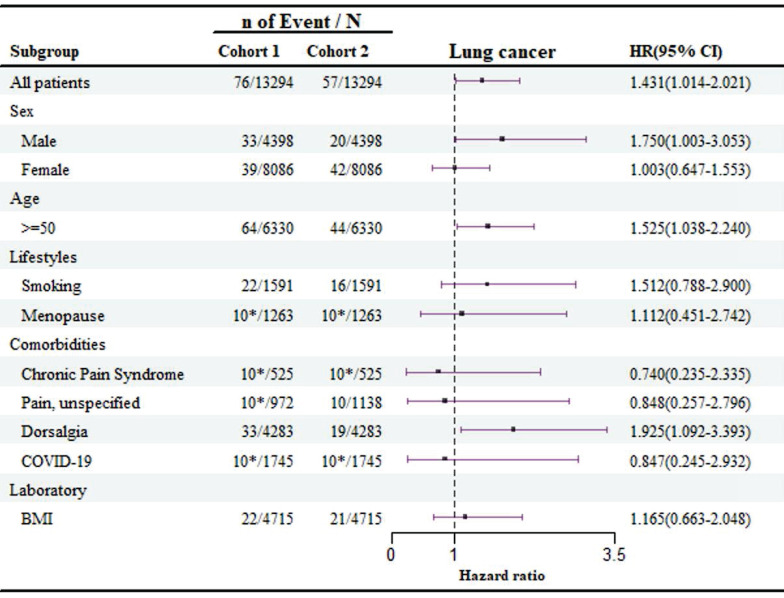
Risk of lung cancer exposed to olfactory and gustatory disturbances compared to non- olfactory and gustatory disturbances in different groups. **Cohort 1** is the group of individuals with disturbances of smell and taste;** Cohort 2** is the group of individuals without disturbances of smell and taste. The privacy policy of TriNetx, where items with a count of 10 or 10* may represent fewer than 10 individuals.

The subgroup analysis of demographic factors, lifestyle habits, and comorbidities related to lung cancer risk is shown in Figure 3. The findings reveal that patients over the age of 50 have a significantly higher risk of developing lung cancer compared to younger individuals (HR: 1.525, 95 % CI: 1.038-2.240), with men being particularly at risk (HR: 1.750, 95 % CI: 1.003-3.053). Moreover, patients with dorsalgia (back pain) were found to have an elevated risk of lung cancer (HR: 1.925, 95 % CI: 1.092-3.393), emphasizing the need for enhanced screening and monitoring for these symptoms.

In the cohort of 1,745 patients infected with COVID-19, fewer than 10 developed lung cancer. As a result, the data suggest that COVID-19 infection did not have a significant impact on lung cancer risk among patients with olfactory and gustatory disorders in the sleep disorder group (HR: 0.847, 95 % CI: 0.245-2.932), indicating a limited effect of COVID-19 in this context.

## Discussion

This study provides valuable insight into the potential role of olfactory and gustatory disturbances as early markers for lung cancer in patients with sleep disorders. The significantly increased risk of lung cancer in patients with sensory dysfunction highlights the need to consider these impairments in clinical evaluations, particularly in high-risk populations such as older adults and males. The results align with prior research indicating that sensory disturbances can be linked to systemic diseases, including malignancies, possibly due to shared underlying mechanisms like inflammation, immune response, and neurological damage.

Our results demonstrate a clear association between olfactory and gustatory disorders and an increased risk of developing lung cancer. With a hazard ratio of 1.431, patients with these sensory disturbances were shown to have a significantly higher likelihood of lung cancer occurrence compared to those without such impairments. This finding supports the hypothesis that olfactory and gustatory dysfunctions may be early indicators of systemic changes related to cancer development, possibly due to shared pathophysiological mechanisms, such as inflammation, neurodegeneration, or immune dysregulation. The Kaplan-Meier curves further reinforce this association, revealing a significant difference in the probability of lung cancer incidence between the two cohorts. Emerging research indicates that olfactory receptors (ORs), traditionally associated with smell, are ectopically expressed in various non-olfactory tissues, including cancerous cells. These receptors can influence tumor cell proliferation, apoptosis, and metastasis, thereby playing a role in tumorigenesis. For instance, specific ORs have been found to modulate pathways that control cell cycle progression and survival, suggesting a direct link between sensory receptors and cancer biology 19. These results underscore the importance of considering sensory dysfunctions in the early detection of lung cancer, particularly in high-risk populations, and suggest that clinicians should closely monitor patients presenting with unexplained olfactory and gustatory disturbances. Further research is needed to explore the underlying biological mechanisms and potential use of sensory impairments as part of lung cancer screening protocols.

The subgroup analysis identifying a higher lung cancer risk in patients with dorsalgia (back pain) is an intriguing finding that warrants further exploration. Dorsalgia is often associated with musculoskeletal or neurological conditions 20, 21, but its potential link to lung cancer risk may indicate that chronic pain could be an early symptom of an underlying malignancy 22, 23. This association could be due to several factors, including the possibility that lung cancer metastasis or tumor growth could cause referred pain to the back or thoracic region 24. Additionally, the chronic inflammatory state 25 seen in both pain syndromes and cancer development may suggest shared biological pathways, such as prolonged immune activation or oxidative stress 26, that contribute to both conditions. Clinically, these findings suggest that patients presenting with dorsalgia, particularly those with other risk factors such as smoking or respiratory symptoms, may benefit from more comprehensive screening for lung cancer 27, 28. Further studies are needed to confirm this link and to clarify whether dorsalgia could serve as an early warning sign of lung cancer or is merely coincidental in these patients.

In the other subgroup analysis showing a higher lung cancer risk in males over 50 years old is particularly interesting, as it contrasts with the common understanding that women over 50 are generally more prone to sleep disorders 29, 30 and olfactory or gustatory disturbances 31, 32, especially during and after menopause. This finding suggests that, despite the higher prevalence of sleep and sensory disorders in older women, men in this age group may experience more severe or clinically significant implications of these disorders, particularly in relation to lung cancer risk. One possible explanation is that men may have higher exposure to known lung cancer risk factors, such as smoking and occupational hazards, which could exacerbate the impact of sensory impairments 33, 34. Additionally, hormonal differences and varying immune responses between men and women could play a role in this increased cancer risk 35, 36. This highlights the importance of not only focusing on women but also considering older men as a high-risk group for lung cancer, particularly if they exhibit symptoms of sensory disturbances. Further research is needed to unravel the gender-specific mechanisms driving these observations and to optimize screening strategies for both men and women.

While this study provides valuable insights into the relationship between olfactory and gustatory disturbances and lung cancer risk in patients with sleep disorders, several limitations must be acknowledged. First, as a retrospective cohort study utilizing data from electronic medical records (EMRs), this research is subject to potential misclassification or incomplete data, particularly concerning the accurate documentation of sensory impairments and comorbid conditions. Additionally, EMR data may lack critical information on lifestyle factors, such as smoking history, alcohol use, or occupational exposures, which are key determinants of lung cancer risk but were not fully captured in this analysis. Second, although propensity score matching was employed to balance observed covariates, unmeasured or inadequately measured confounders could still influence the results. Employing quantitative bias analysis methods, such as probabilistic bias analysis, could help evaluate the impact of residual confounding on the findings. Variables such as socioeconomic status or environmental exposures, which may simultaneously affect sensory dysfunction and lung cancer risk, were not included in the dataset, potentially influencing the observed associations. Third, the generalizability of these findings may be limited, as the study population predominantly consisted of individuals with sleep disorders. This specific cohort may not adequately represent the broader population at risk for lung cancer, and the results may not be fully applicable to individuals without sleep disturbances. Fourth, the sample size for certain subgroups, particularly those assessing the impact of COVID-19 infection, was limited. This constraint may reduce the statistical power to detect significant associations and limit the generalizability of the findings. Future studies with larger sample sizes are essential to validate the subgroup analyses. Lastly, while the study identified a higher lung cancer risk in patients with dorsalgia and in males over 50 years old, the relatively small sample sizes in these subgroups and the lack of in-depth exploration of the biological mechanisms linking these factors to lung cancer warrant further investigation. Prospective studies are needed to confirm these associations and provide a clearer understanding of the underlying pathophysiological pathways.

Specifically, we propose that routine screening for olfactory and gustatory disturbances be integrated into clinical assessments for individuals at high risk for lung cancer, particularly those with sleep disorders. Sensory dysfunctions could serve as a cost-effective and non-invasive marker for identifying patients who may benefit from more targeted diagnostic evaluations. Additionally, we suggest incorporating sensory impairment assessments into existing cancer risk stratification models to enhance their predictive accuracy. This could be particularly valuable for older adults and males with sleep disorders, as these groups were identified as having elevated risks in our study. By emphasizing these applications, we aim to bridge the gap between research findings and real-world clinical utility, ensuring that the results of this study contribute to improved early detection strategies and patient outcomes.

## Conclusion

Olfactory and gustatory disturbances may serve as early markers for lung cancer, particularly in older individuals and males with sleep disorders. These findings highlight the potential for incorporating sensory assessments into routine clinical screenings for high-risk populations, offering a new avenue for early lung cancer detection. Future studies should explore the biological mechanisms underlying this association and consider larger, prospective cohorts to validate these findings. Additionally, further research is needed to clarify the role of COVID-19 in sensory dysfunction and lung cancer risk.

## Supplementary Material

Supplementary tables.

## Figures and Tables

**Table 1:** Baseline characteristics of study subjects (before and after Propensity score matching).

	Before matching				After matching		
	Cohort 1 (n = 13,294)	Cohort 2 (n = 1,113,235)	Std diff		Cohort 1 (n = 13,294)	Cohort 2 (n = 13,294)	Std diff
**Age at index**							
Mean ± SD	54.6±16.3	52.2±17.5	**0.1411**		54.6±16.3	54.8±16.5	0.0117
**Gender, n(%)**							
Female	8228(61.9%)	626459(56.3%)	**0.1145**		8228(61.9%)	8131(61.2%)	0.0150
Male	4415(33.2%)	424318(38.1%)	**0.1025**		4415(33.2%)	4490(33.8%)	0.0120
Unknown Gender	651(4.9%)	62458(5.6%)	0.0320		651(4.9%)	673(5.1%)	0.0076
**Race, n(%)**							
White	9234(69.5%)	760028(68.3%)	0.0257		9234(69.5%)	9308(70.0%)	0.0121
Black or African American	1587(11.9%)	124548(11.2%)	0.0234		1587(11.9%)	1523(11.5%)	0.0150
Asian	378(2.8%)	34824(3.1%)	0.0167		378(2.8%)	403(3.0%)	0.0111
Unknown Race	1515(11.4%)	146566(13.2%)	0.0539		1515(11.4%)	1508(11.3%)	0.0017
Other Race	431(3.2%)	33995(3.1%)	0.0108		431(3.2%)	396(3.0%)	0.0152
**Lifestyles, n(%)**							
Nicotine dependence	1246(9.4%)	83818(7.5%)	0.0663		1246(9.4%)	1178(8.9%)	0.0178
Tobacco use	426(3.2%)	29128(2.6%)	0.0350		426(3.2%)	442(3.3%)	0.0068
Alcohol related disorders	377(2.8%)	29730(2.7%)	0.0101		377(2.8%)	391(2.9%)	0.0063
**Comorbidities, n(%)**							
Anxiety disorders	4573(34.4%)	247180(22.2%)	**0.2732**		4573(34.4%)	4568(34.4%)	0.0008
Depressive episode	3362(25.3%)	177083(15.9%)	**0.2336**		3362(25.3%)	3317(25.0%)	0.0078
Overweight and obesity	3021(22.7%)	167707(15.1%)	**0.1966**		3021(22.7%)	2955(22.2%)	0.0119
Diabetes mellitus	2333(17.5%)	146788(13.2%)	**0.1212**		2333(17.5%)	2230(16.8%)	0.0205
COVID-19	1097(8.3%)	27960(2.5%)	**0.2565**		1097(8.3%)	1091(8.2%)	0.0016
Chronic kidney disease	969(7.3%)	54945(4.9%)	0.0984		969(7.3%)	933(7.0%)	0.0105
Cerebrovascular diseases	795(6.0%)	49894(4.5%)	0.0673		795(6.0%)	741(5.6%)	0.0174
Heart failure	757(5.7%)	48607(4.4%)	0.0608		757(5.7%)	689(5.2%)	0.0226
COPD	720(5.4%)	47787(4.3%)	0.0523		720(5.4%)	681(5.1%)	0.0131
Chronic pain syndrome	524(3.9%)	23067(2.1%)	**0.1096**		524(3.9%)	486(3.7%)	0.0150
Other peripheral vascular diseases	406(3.1%)	24969(2.2%)	0.0505		406(3.1%)	380(2.9%)	0.0115
Acute myocardial infarction	220(1.7%)	14318(1.3%)	0.0306		220(1.7%)	196(1.5%)	0.0145
Liver disease, unspecified	192(1.4%)	8183(0.7%)	0.0684		192(1.4%)	173(1.3%)	0.0123
Rheumatoid arthritis with rheumatoid factor	112(0.8%)	5644(0.5%)	0.0410		112(0.8%)	94(0.7%)	0.0154
Peptic ulcer, site unspecified	54(0.4%)	3273(0.3%)	0.0190		54(0.4%)	47(0.4%)	0.0086
Alzheimer's disease	46(0.3%)	6643(0.6%)	0.0366		46(0.3%)	50(0.4%)	0.0050
Paraplegia	11(0.1%)	2020(0.2%)	0.0272		11(0.1%)	21(0.2%)	0.0217
**Medical utilization, n(%)**							
Office or Other Outpatient Services	9777(73.5%)	632987(56.9%)	**0.3558**		9777(73.5%)	9835(74.0%)	0.0099
Emergency Department Services	3164(23.8%)	183903(16.5%)	**0.1822**		3164(23.8%)	3084(23.2%)	0.0142
Preventive Medicine Services	2334(17.6%)	142252(12.8%)	**0.1335**		2334(17.6%)	2401(18.1%)	0.0132
Hospital Inpatient and Observation Care Services	1423(10.7%)	91738(8.2%)	0.0842		1423(10.7%)	1290(9.7%)	0.0331
**Medical utilization, n(%)**							
fentanyl	2546(19.2%)	132611(11.9%)	**0.2009**		2546(19.2%)	2462(18.5%)	0.0162
oxycodone	1925(14.5%)	112827(10.1%)	**0.1326**		1925(14.5%)	1823(13.7%)	0.0221
morphine	959(7.2%)	56850(5.1%)	0.0877		959(7.2%)	869(6.5%)	0.0268
codeine	787(5.9%)	42993(3.9%)	0.0955		787(5.9%)	773(5.8%)	0.0045
**Laboratory**							
BMI							
≥ 30 kg/m2	4726(35.6%)	302979(27.2%)	**0.1803**		4726(35.6%)	4748(35.7%)	0.0035
Prostate specific Ag [Mass/volume] in Serum or Plasma							
≥ 10 ng/mL	23(0.2%)	1451(0.1%)	0.0110		23(0.2%)	19(0.1%)	0.0076
Carcinoembryonic Ag [Mass/volume] in Serum or Plasma							
> 30 mm/h	60(0.5%)	1980(0.2%)	0.0488		60(0.5%)	46(0.3%)	0.0167
Cancer Ag 19-9 [Units/volume] in Serum or Plasma							
≥ 37 U/mL	44(0.3%)	1434(0.1%)	0.0422		44(0.3%)	38(0.3%)	0.0081
Cancer Ag 125 [Units/volume] in Serum, Plasma or Blood							
≥ 35 U/mL	32(0.2%)	1281(0.1%)	0.0298		32(0.2%)	27(0.2%)	0.0080
Choriogonadotropin.beta subunit [Units/volume] in Serum, Plasma or Blood							
≥ 25 mIU/mL	26(0.2%)	1377(0.1%)	0.0180		26(0.2%)	25(0.2%)	0.0017
Alpha-1-Fetoprotein [Mass/volume] in Serum, Plasma or Blood							
≥ 20 ng/mL	10(0.1%)	1130(0.1%)	0.0088		10(0.1%)	10(0.1%)	0.0000
Cancer Ag 15-3 [Units/volume] in Serum or Plasma							
> 30 U/mL	10(0.1%)	287(0.0%)	0.0220		10(0.1%)	10(0.1%)	0.0000

After matching in Table 1 is conducted based on Age at Index, gender, race, lifestyle, comorbidities, socioeconomic status, medical utilization. **Cohort 1** is the group which individuals with disturbances of smell and taste; **Cohort 2** is the group which individuals without disturbances of smell and taste. The privacy policy of TriNetx, where items with a count of 10 or 10* may represent fewer than 10 individuals.

**Table 2:** Incidence of outcomes in individuals with and without olfactory or gustatory dysfunction following propensity score matching.

Outcome	Patients with outcome/population at risk		Hazard ratio (95%CI)
	Cohort 1	Cohort 2	
**Lung cancer**	76/13294	57/13294	1.431(1.014, 2.021)

The p-value of Proportionality is 0.8038 CI means confidence interval. **Cohort 1** is the group which individuals with disturbances of smell and taste; **Cohort 2** is the group which individuals without disturbances of smell and taste.
