# Supplementary material for: Olfactory and Gustatory Disturbances as Early Indicators of Lung Cancer in Patients with Sleep Disorders: A Retrospective Cohort Study from the TriNetX US Collaborative Networks
Source: Int J Med Sci. 2025 Jan 13;22(3):723–31. doi: 10.7150/ijms.106014 (PMC11783070; doi:10.7150/ijms.106014)
Supplement: Supplementary file 1 — Supplementary tables. [file ijmsv22p0723s1.pdf]

Table S1. Codes that define sleep disorder.

Code	Description
ICD10CM: G47.01	Insomnia due to medical condition
ICD10CM: G47.09	Other insomnia
ICD10CM: G47.20	Circadian rhythm sleep disorder, unspecified type
ICD10CM: G47.21	Circadian rhythm sleep disorder, delayed sleep phase type
ICD10CM: G47.22	Circadian rhythm sleep disorder, advanced sleep phase type
ICD10CM: G47.23	Circadian rhythm sleep disorder, irregular sleep wake type
ICD10CM: G47.24	Circadian rhythm sleep disorder, free running type
ICD10CM: G47.25	Circadian rhythm sleep disorder, jet lag type
ICD10CM: G47.26	Circadian rhythm sleep disorder, shift work type
ICD10CM: G47.27	Circadian rhythm sleep disorder in conditions classified elsewhere
ICD10CM: G47.29	Other circadian rhythm sleep disorder
ICD10CM: G47.5	Parasomnia
ICD10CM: G47.8	Other sleep disorders
ICD10CM: G47.9	Sleep disorder, unspecified
ICD10CM: F51.01	Primary insomnia
ICD10CM: F51.8	Other sleep disorders not due to a substance or known physiological condition
ICD10CM: F51.9	Sleep disorder not due to a substance or known physiological condition, unspecified

Table S2. Codes that define neoplasm (Head & Neck cancer).

Code	Description
ICD10CM: C10	Malignant neoplasm of oropharynx
ICD10CM: C11	Malignant neoplasm of nasopharynx
ICD10CM: C13	Malignant neoplasm of hypopharynx
ICD10CM: C14.0	Malignant neoplasm of pharynx, unspecified
ICD10CM: C14.8	Malignant neoplasm of overlapping sites of lip, oral cavity and pharynx
ICD10CM: C31.8	Malignant neoplasm of overlapping sites of accessory sinuses
ICD10CM: C32.8	Malignant neoplasm of overlapping sites of larynx
ICD10CM: C41.0	Malignant neoplasm of bones of skull and face
ICD10CM: C49.0	Malignant neoplasm of connective and soft tissue of head, face and neck
ICD10CM: C76.0	Malignant neoplasm of head, face and neck
ICD10CM: D02.0	Carcinoma in situ of larynx
ICD10CM: D00.00	Carcinoma in situ of oral cavity, unspecified site Carcinoma in situ of oral cavity, unspecified site
ICD10CM: D00.08	Carcinoma in situ of pharynx
ICD10CM: D14.0	Benign neoplasm of middle ear, nasal cavity and accessory sinuses
ICD10CM: D14.1	Benign neoplasm of larynx
ICD10CM: D21.0	Benign neoplasm of connective and other soft tissue of head, face and neck
ICD10CM: D36.7	Benign neoplasm of other specified sites
ICD10CM: D49.1	Neoplasm of unspecified behavior of respiratory system
ICD10CM: D49.2	Neoplasm of unspecified behavior of bone, soft tissue, and skin
ICD10CM: D49.89	Neoplasm of unspecified behavior of other specified sites

Table S3. Codes that define disturbances of smell and taste.

Code	Description
ICD10CM: R43	Disturbances of smell and taste
ICD10CM: R43.0	Anosmia
ICD10CM: R43.1	Parosmia
ICD10CM: R43.2	Parageusia
ICD10CM: R43.8	Other disturbances of smell and taste
ICD10CM: R43.9	Unspecified disturbances of smell and taste
